# Dietary knowledge, attitude, practice, and associated factors among pregnant mothers in Ethiopia: a systematic review and meta-analysis

**DOI:** 10.3389/fpubh.2024.1393764

**Published:** 2024-09-11

**Authors:** Ewunetie Mekashaw Bayked, Ebrahim M. Yimer, Tiruset Gelaw, Abdu Seid Mohammed, Nigusie Abebaw Mekonen

**Affiliations:** ^1^Department of Pharmacy, College of Medicine and Health Sciences (CMHS), Wollo University, Dessie, Ethiopia; ^2^Department of Midwifery, College of Medicine and Health Sciences (CMHS), Wollo University, Dessie, Ethiopia

**Keywords:** diet, nutrition, knowledge, attitude, practice, factor, pregnancy, Ethiopia

## Abstract

**Background:**

Despite global efforts, progress in reducing maternal malnutrition falls short of international goals, which is the same for Ethiopia, provided that studying dietary knowledge, attitude, and practice and their determinants is crucial to developing and implementing effective interventions, which this review tried to investigate in an Ethiopian context.

**Methods:**

We searched on Scopus, HINARI, PubMed, and Google Scholar on January 3, 2024. We used the Joanna Briggs Institute’s (JBI’s) tools and the “preferred reporting items for systematic reviews and meta-analyses (PRISMA) 2020 statement” to evaluate bias and frame the review, respectively. The data were analyzed using Stata 17. Certainty was assessed using sensitivity and subgroup analyses and the Luis Furuya-Kanamori (LFK) index. The random effects model was used to determine the effect estimates with a *p* value less than 0.05 and a 95% CI.

**Results:**

The pooled good dietary knowledge, favorable attitude, and good practice were 48.0% (95% CI: 39.0–57.0%), 47.0% (95% CI: 38.0–55.0%), and 34.0% (95% CI: 28.0–40.0%), respectively. Knowledge and attitude had bidirectional relationships and were affected by sociodemographic variables and gynecological issues. The dietary practice was influenced by urban residency (OR = 6.68, 95% CI: 2.49–10.87), food security (OR = 3.51, 95% CI: 1.02–5.99), knowledge (OR = 4.53, 95% CI: 3.22–5.74), nutrition information (OR = 3.07, 95% CI: 1.13–5.02), attitude (OR = 2.32, 95% CI: 1.34–3.30), family support (OR = 2.14, 95% CI: 1.43–2.85), perceived severity of malnutrition (OR = 2.07, 95% CI: 1.82–2.31), and positive perception of dietary benefit (OR = 2.19, 95% CI: 1.56–2.82).

**Conclusion:**

The good dietary practice was lower than the knowledge and the favorable attitude toward it. It was influenced by sociodemographic variables, income and wealth, knowledge and information, attitudes and intentions, gynecological and illness experiences, family support and decision-making, and expectations of nutrition outcomes and habits. Sociodemographic and gynecological issues were also found to influence both dietary knowledge and attitude, which were also found to have bidirectional relationships.

**Systematic review registration**: PROSPERO identifier: CRD42023440688.

## Introduction

Health is a fundamental human right that relies on consuming meals with essential nutrients for survival and thriving (1). Good health is a crucial outcome of development and is valuable in itself (2), and good nutrition is crucial for realizing this and achieving universal health coverage (UHC) (3). Good nutrition involves obtaining the right amount of essential nutrients, such as carbohydrates, fats, fiber, minerals, proteins, vitamins, and water (4). However, the global burden of undernutrition remains high, with millions facing starvation and malnutrition, particularly women and children (5). These population groups are most vulnerable to malnutrition due to low dietary intake, inequitable food distribution, and high costs of pregnancy and lactation (6). This is because nutritional requirements increase during pregnancy to support the developing fetus and further rise during lactation to aid in the growth and development of the newborn (7).

Pregnancy necessitates increased energy and protein intake, with an additional 150 kcal/day in the first trimester and 350 kcal/day in the second and third trimesters. Up to 20 g of protein daily is required for maternal and fetal tissue synthesis (8). Vitamin and mineral intake also increases, with iron requirements rising from 21 to 35 mg/day. Dietary sources include liver, meat, fish, poultry, and vegetables. Adult women need 600 mg/day of calcium, while the fetus requires 1,200 mg/day; vitamin D is crucial for calcium metabolism (7). Zinc requirements increase from 10 to 15 mg/day. Water-soluble vitamins, such as thiamin, riboflavin, niacin, vitamin B_6_, and B_12_, are essential during pregnancy (9). Folic acid needs to be increased to 500 μg during pregnancy to prevent low birth weight, preterm birth, and congenital malformations. Vitamin C needs increase by 20 mg and can be obtained from fresh fruits, citrus fruits, green leafy vegetables, cabbage, guava, and germinating pulses (7).

Poor maternal nutrition is a significant global issue (10, 11). Over 1 billion women worldwide face malnutrition, with low- and middle-income countries (LMICs) bearing the highest burden (12). Undernutrition is prevalent among the majority of women of reproductive age in most developing countries (11), particularly Sub-Saharan Africa (SSA), which faces significant nutritional insecurity due to poor infrastructure, limited resources, conflict, HIV, and limited access to health services (13). Ethiopia is a country in the SSA greatly affected by this issue. For instance, the prevalence of undernutrition among pregnant mothers in Afar (14), Southern Ethiopia (15), Oromia (16), and Western Ethiopia (17) was 30.9, 41.2, 44.9, and 39.2%, respectively.

The nutritional status of pregnant and breastfeeding women is vital for their own health and that of future generations (18). Poor nutrition during and after pregnancy can result in adverse effects for both the mother and her baby (19), including increased risks of maternal mortality, morbidity, and reduced productivity due to undernourishment and anemia (20). Malnutrition in women, caused by inadequate diets, care services, and practices, heightens their risk of illness, death, and adverse pregnancy outcomes. This contributes to undernutrition in early childhood, which impacts school readiness, enrollment, and learning performance. Consequently, this can result in poverty during adulthood due to restricted employment opportunities and reduced productivity, thus continuing the cycle of malnutrition across generations. However, global progress in reducing malnutrition among women of reproductive age falls short of international goals and targets. Addressing these issues is crucial for ensuring a healthy and prosperous future for all (21).

Inadequate diets deficient in essential nutrients such as iodine, iron, folate, calcium, and zinc may result in conditions such as anemia, pre-eclampsia, hemorrhage, and maternal mortality. Additionally, they can contribute to adverse outcomes for infants, including stillbirth, low birth weight, malnutrition, and developmental delays (22). Twenty percent of maternal deaths in Africa are due to anemia, primarily caused by iron and folate deficiencies, with contributing factors including chronic energy deficiency and poor micronutrient status (23). Evidence shows that iron deficiency is the most common micronutrient deficiency in pregnancy, affecting 40% of pregnancies globally. The highest prevalence is in Southeast Asia (49%), followed by Africa (46%), and the Eastern Mediterranean (41%). The Western Pacific (33%), the Americas (26%), and Europe (27%) have lower prevalence (24).

Barriers to adequate dietary intake in pregnant and lactating women, influenced by personal preferences, cultural beliefs, economic constraints, and perceptions of food appropriateness (25). Psychosocial factors like anxiety, depression, anger, fatigue, social support, and stress can increase macronutrient consumption while decreasing micronutrient intake, requiring consideration in pregnancy diet counseling (26). Providing a combination of micronutrients proves to be a safe and effective approach for enhancing maternal nutritional well-being during pregnancy. This strategy exhibits superior advantages in reducing anemia and preventing low birth weight compared to solely administering iron and folic acid (27). Maternal nutrition encompasses women’s nutritional needs during pregnancy, breastfeeding, and pre-conception periods, focusing on essential micronutrients like iron, vitamin B12, folic acid, vitamin D, and selenium (20).

Good nutrition is crucial for optimal growth, development, and health, reducing chronic diseases such as heart disease, stroke, diabetes, and cancer. Food is intrinsically linked to human existence, providing social benefits and survival, unlike other commodities (28). It is particularly important for women’s overall health as it impacts their children and future generations, leading to economic, humanitarian, and health consequences for society (29). This dictates that nutrition counseling is essential for prenatal care because it affects a woman’s health, pregnancy outcomes, and the health of her fetus-neonate (30). Nutritional care encompasses the provision of nutrition, nutrient delivery, and education for meal service, as well as treating nutrition-related conditions, including preventive and clinical nutrition (31).

Hence, nutrition-related studies focusing on evaluating people’s knowledge, attitudes, and practices regarding diet and health may guide to develop intervention design and strategies to assess the outcomes of those interventions. These studies can offer insights into the social, psychological, and behavioral determinants of nutritional status, helping to better understand specific situations (32). However, to the best of our knowledge, there is no nationally pooled data in Ethiopia regarding the dietary knowledge, attitude, and practices of pregnant mothers. Therefore, the purpose of this systematic review and meta-analysis was to determine dietary knowledge, attitudes, practices, and associated factors among pregnant mothers in Ethiopia. Accordingly, the main question to be answered by the review, using the CoCoPop Framework (33)—Condition (knowledge, attitude, and practice), Context (Ethiopia), and Population (pregnant mothers)—was: what is the prevalence of dietary knowledge, attitudes, and practices among pregnant women in Ethiopia?

## Methods

### Registration and protocol

The protocol for this review was registered on PROSPERO, accessible at CRD42023440688. We used the “PRISMA 2020 Statement: An Updated Guideline for Reporting Systematic Reviews” as a framework (34) (Supplementary material 1). However, to pictorially present the screening process of the studies, we used the PRISMA 2009 flow diagram (35) because of its ease and clarity, while we discussed the screening process in words in accordance with the PRISMA 2020 flow diagram.

### Eligibility criteria

All original and published cross-sectional studies reporting pregnant mothers’ dietary knowledge, attitudes, and practices and/or factors influencing them were deemed eligible for the systematic review. Studies conducted in both community and institutional settings were considered. The selection of studies was based on several parameters, including outcome variables, study population, year of the study, regional context, sample size, and response rate.

### Information sources and search strategy

We searched Scopus, HINARI, PubMed, and Google Scholar on January 3, 2024, using searching terms such as “dietary” OR “diet” OR “nutrition” OR “nutrient” AND “practice” OR “knowledge” OR “attitude” AND “factor” AND “pregnant” OR “pregnancy” OR “mother” AND “Ethiopia” (Supplementary material 2). Manual searches were performed on PubMed and HINARI. Conversely, Scopus and Google Scholar were searched using the “Publish or Perish” database searching tool, version 8 (36).

### Selection process

After excluding duplicate studies with EndNote 20, EMB and TT independently screened the remaining studies. The selection process was meticulously conducted by these researchers. Initially, articles were refined based on their title and abstract; subsequently, full-text revisions were performed independently and then collaboratively until a consensus was reached. In cases of disagreement, a third reviewer was consulted for resolution.

### Data collection process and data items

A Microsoft Excel 2019 spreadsheet was used for data extraction. The outcome variables—population (study units), year of study, context, sample size, response rate, and proportions—were extracted using this spreadsheet. Two independent reviewers, EB and TG, extracted the data, compared their findings, and reached a consensus. In cases where agreement could not be reached, a third reviewer was invited to help achieve consensus. The primary outcomes of this systematic review and meta-analysis were dietary knowledge, attitude, and practice among pregnant mothers. Additional outcomes included other factors influencing the primary outcome variables.

#### Study risk of bias assessment

Two reviewers, EB and TG, independently assessed the risk of bias in the included studies using tools developed by the Joanna Briggs Institute (JBI), which comprise eight criteria. The assessment focused on several aspects: inclusion in the sample, descriptions of study subjects and settings, validity and reliability of measurements, confounding factors and strategies to address them, and appropriateness of the outcome measures. Scores of 7 or higher were classified as low risk, 5–6 as medium risk, and 4 or lower as high risk. Studies identified as low- and medium-risk were then included in the review. All inconsistencies were addressed through discussion and, if necessary, the involvement of a third reviewer.

#### Effect measures and synthesis methods

For the qualitative synthesis, thematic strategies were utilized to categorize the outcome variables conceptually. Preliminary effect measures for the quantitative synthesis were calculated based on the qualitative synthesis using a Microsoft Excel 2019 spreadsheet. STATA 17 was employed to determine the effect estimates (proportions and odds ratios—ORs) of dietary knowledge, attitude, and practice. We included the associated variables, which were dichotomized regardless of their significance levels. Subgroup analyses were subsequently conducted to compare these effect estimates across studies focusing on the outcome variables. The overall level of statistical significance was set at a *p* value of less than 0.05 with a 95% confidence interval (CI).

#### Reporting bias and certainty assessment

Heterogeneity between studies was assessed using the I2 statistic. The influence of each study on the overall meta-analysis was measured using percentages of weights and subgroup analysis, comparing the effect estimates (proportions and ORs) of dietary knowledge, attitude, and practice between regions and based on study settings. A sensitivity analysis was also conducted to determine the outlier studies. Moreover, Doi plots were used to examine potential inter-study bias.

## Results

### Study selection

**Figure 1:**
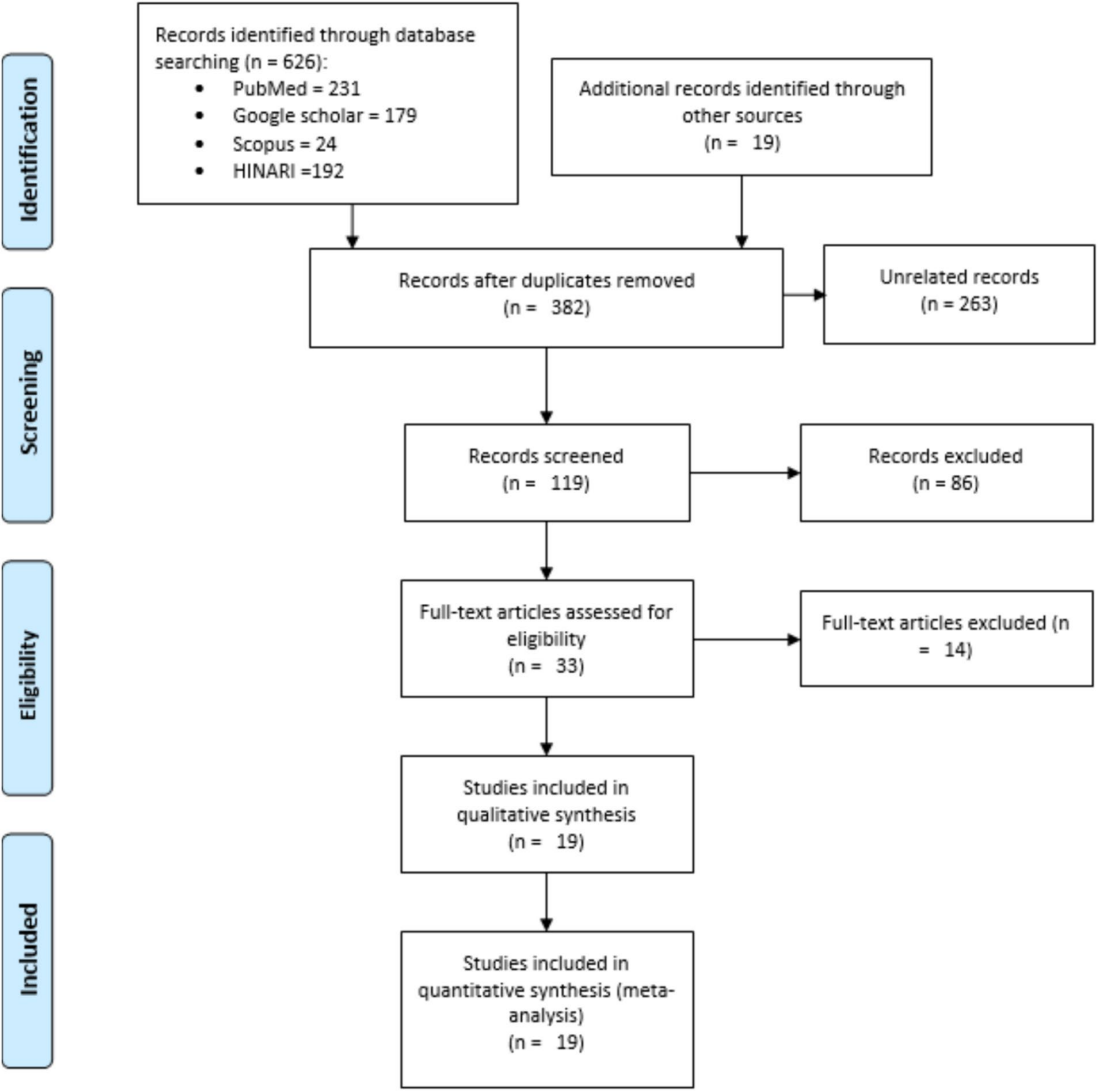
Schematic description of the screening processes for literature, 2024.

We systematically searched Google Scholar (*n* = 179), HINARI (*n* = 192), Scopus (*n* = 24), and PubMed (*n* = 231) on January 3, 2024. Additionally, 19 records were found through other sources, resulting in a total of 645 resources (Figure 1). Following the removal of duplicates, 382 articles remained. Then, after excluding 263 resources through relevance, 119 articles were screened for title and abstract evaluation, which resulted in the exclusion of 86 resources to retain 33 papers. After a thorough examination of the full texts, 14 studies were excluded due to reasons such as being preprints (37–40), having outcomes incongruent with the objective or title (41–43), being unpublished (44, 45), or exhibiting a high risk of bias (46–49). Finally, 19 resources were identified for inclusion, all of which were considered suitable for the quantitative meta-analysis.

### Risk of bias in studies

**Figure 2:**
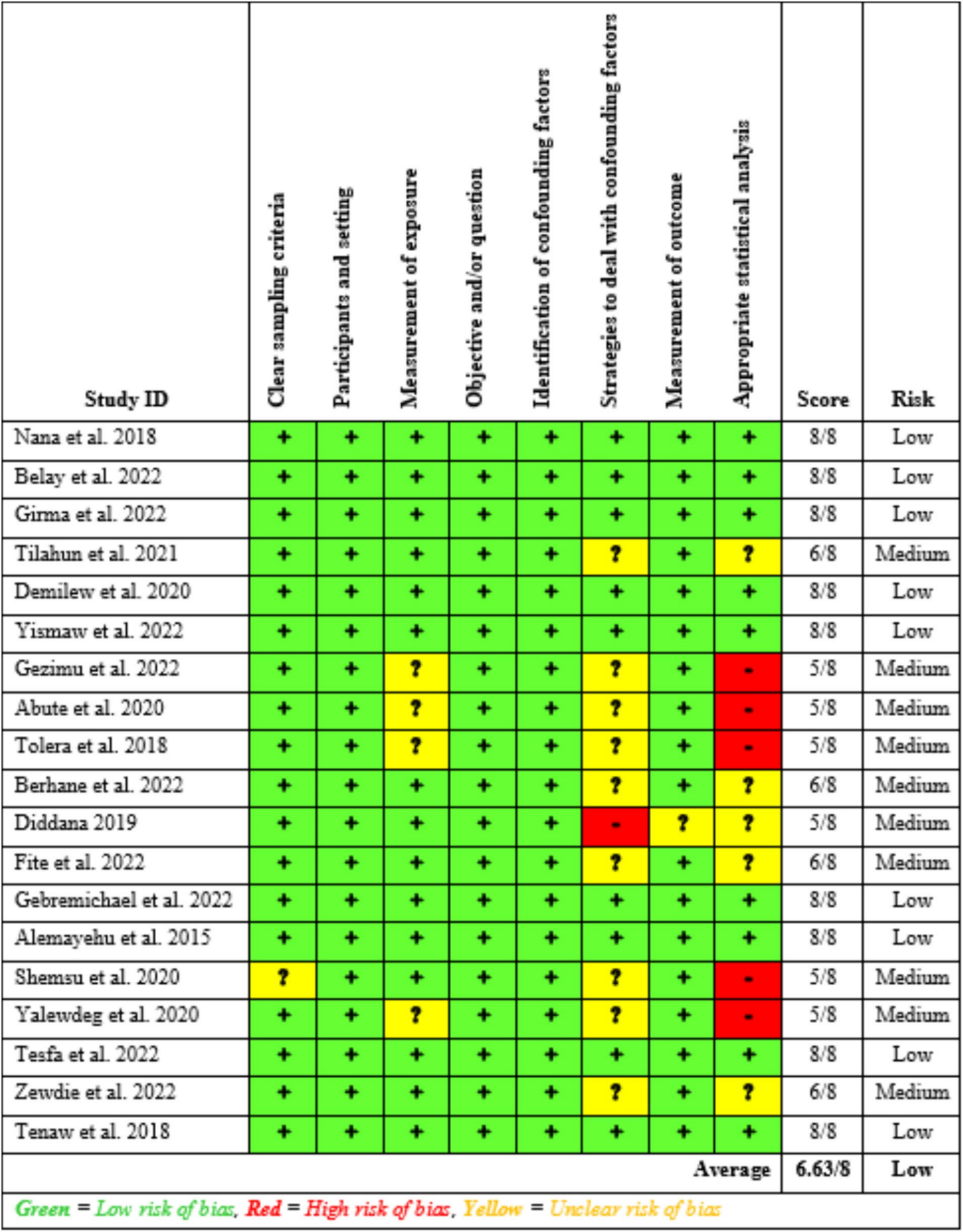
Summary of the risk of bias assessment of the included studies (*n* = 19), 2024.

The risk of bias in the included studies was evaluated using JBI’s critical appraisal tool. Subsequently, studies with low and medium risk were incorporated into the review. Figure 2 depicts that the average risk of bias across the studies was 6.63, representing 82.9%.

### Qualitative synthesis

#### Studies and participants

A total of 19 studies from five regions of Ethiopia have been included (Tables 1, 2), from southern nationalities and people’s regions (SNNPR, *n* = 6), Oromia (*n* = 5), Amhara (*n* = 5), Addis Ababa (*n* = 2), and Harar (*n* = 1). Data were synthesized regarding the pregnant mothers’ dietary knowledge, attitude, and practice. Among the 19 studies, the majority (*n* = 10) were conducted in community settings, while the remainder (*n* = 9) were carried out at health facilities.

**Table 1:** Characteristics of individual studies pertaining the dietary knowledge and attitude of pregnant mothers.

Characteristics of individual studies pertaining the dietary knowledge of pregnant mothers:								
Study ID	Design	Setting	PY	SY	Region	SS	RR	GK
Nana et al. (50)	Cross-Sectional	Community	2018	2016	Amhara	616	616	242
Belay et al. (51)	Cross-Sectional	Community	2022	2021	Amhara	615	615	167
Girma et al. (52)	Cross-Sectional	Institution	2022	2021	SNNPR	568	566	134
Tilahun et al. (53)	Cross-Sectional	Institution	2021	2021	SNNPR	378	374	94
Demilew et al. (38)	Cross-Sectional	Community	2020	2018	Amhara	712	694	138
Yismaw et al. (54)	Cross-Sectional	Community	2022	2019	Oromia	610	592	185
Gezimu et al. (55)	Cross-Sectional	Institution	2022	2021	SNNPR	378	378	198
Abute et al. (56)	Cross-Sectional	Community	2020	2019	SNNPR	618	618	181
Gebremichael et al. (57)	Cross-Sectional	Community	2022	2018	Oromia	770	750	250
Alemayehu et al. (58)	Cross-Sectional	Community	2015	2014	Amhara	580	574	303
Shemsu et al. 2020 (59)	Cross-Sectional	Institution	2020	2018	Oromia	378	378	79
Yalewdeg et al. (60)	Cross-Sectional	Institution	2020	2018	SNNPR	351	351	160
Tesfa et al. (61)	Cross-Sectional	Institution	2022	2021	Addis Ababa	363	352	268
Tenaw et al. (62)	Cross-Sectional	Institution	2018	2015	Addis Ababa	340	322	87
Aggregate						7,277	7,180	3,366
**Characteristics of individual studies pertaining the dietary attitude of pregnant mothers:**								
**Study ID**	**Design**	**Setting**	**PY**	**SY**	**Region**	**SS**	**RR**	**FA**
Girma et al. (52)	Cross-Sectional	Institution	2022	2021	SNNPR	568	566	134
Tilahun et al. (53)	Cross-Sectional	Institution	2021	2021	SNNPR	378	374	94
Demilew et al. (38)	Cross-Sectional	Community	2020	2018	Amhara	712	694	138
Gezimu et al. (55)	Cross-Sectional	Institution	2022	2021	SNNPR	378	378	198
Gebremichael et al. (57)	Cross-Sectional	Community	2022	2018	Oromia	770	750	235
Alemayehu et al. (58)	Cross-Sectional	Community	2015	2014	Amhara	580	574	331
Yalewdeg et al. (60)	Cross-Sectional	Institution	2020	2018	SNNPR	351	351	150
Tenaw et al. (62)	Cross-Sectional	Institution	2018	2015	Addis Ababa	340	322	156
Aggregate						4,077	4,009	1,807

FA, Favorable attitude; GK, Good knowledge; PY, Publication year; RR, Response rate; SS, Sample size; SY, Study year.

**Table 2:** Characteristics of individual studies pertaining the dietary practice of pregnant mothers.

Study ID	Design	Setting	PY	SY	Region	SS	RR	GP
Nana et al. (50)	Cross-Sectional	Community	2018	2016	Amhara	616	616	242
Belay et al. (51)	Cross-Sectional	Community	2022	2021	Amhara	615	615	167
Girma et al. (52)	Cross-Sectional	Institution	2022	2021	SNNPR	568	566	134
Tilahun et al. (53)	Cross-Sectional	Institution	2021	2021	SNNPR	378	374	94
Demilew et al. (38)	Cross-Sectional	Community	2020	2018	Amhara	712	694	138
Yismaw et al. (54)	Cross-Sectional	Community	2022	2019	Oromia	610	592	185
Gezimu et al. (55)	Cross-Sectional	Institution	2022	2021	SNNPR	378	378	198
Abute et al. (56)	Cross-Sectional	Community	2020	2019	SNNPR	618	618	181
Tolera et al. (63)	Cross-Sectional	Community	2018	2018	Oromia	343	338	91
Berhane et al. (64)	Cross-Sectional	Institution	2022	-	Harar	276	276	80
Diddana (65)	Cross-Sectional	Community	2019	2017	Amhara	604	604	273
Fite et al. (66)	Cross-Sectional	Community	2022	2021	Oromia	475	448	68
Gebremichael et al. (57)	Cross-Sectional	Community	2022	2018	Oromia	770	750	205
Alemayehu et al. (58)	Cross-Sectional	Community	2015	2014	Amhara	580	574	230
Shemsu et al. (59)	Cross-Sectional	Institution	2020	2018	Oromia	378	378	85
Yalewdeg et al. (60)	Cross-Sectional	Institution	2020	2018	SNNPR	351	351	113
Tesfa et al. (61)	Cross-Sectional	Institution	2022	2021	Addis Ababa	363	352	232
Zewdie et al. (67)	Cross-Sectional	Institution	2022	2020	SNNPR	402	392	136
Tenaw et al. (62)	Cross-Sectional	Institution	2018	2015	Addis Ababa	340	322	111
Aggregate						9,377	9,238	3,063

GP, Good practice; PY, Publication year; RR, Response rate; SS, Sample size; SY, Study year.

##### Dietary knowledge

Fourteen included studies reported on mothers’ knowledge of maternal dietary practices during pregnancy (Table 1). The total number of pregnant mothers sampled by these studies was 7,277, with 2,523 or 34.7% in Amhara, 2,293 or 31.5% in SNNPR, 1,758 or 24.2% in Oromia, and 703 or 9.7% in Addis Ababa. Of these, 7,180 or 98.7% participated: 2,499 or 34.8% in Amhara, 2,287 or 31.9% in SNNPR, 1,720 or 24.0% in Oromia, and 674 or 9.4% in Addis Ababa.

##### Dietary attitude

Eight of the included studies reported data on the attitudes of pregnant mothers toward maternal dietary practices during pregnancy (Table 1). A total of 4,077 participants were sampled (1,675 or 41.1% in SNNPR, 1,292 or 31.7% in Amhara, 770 or 18.9% in Oromia, and 340 or 8.3% in Addis Ababa), while 4,009 or 98.3% participated (1,669 or 41.6% in SNNPR, 1,268 or 31.6% in Amhara, 750 or 18.7% in Oromia, and 322 or 8.0% in Addis Ababa).

##### Dietary practice

All 19 included studies reported on the dietary practices of participants (Table 2). A total of 9,377 pregnant mothers were sampled across the studies, with 33.3% (3,127 mothers) from Amhara, 28.7% (2,695 mothers) from SNNPR, 27.5% (2,576 mothers) from Oromia, 7.5% (703 mothers) from Addis Ababa, and 2.9% (276 mothers) from Harar. Of these, 9,238 participated in the studies: 33.6% (3,103 mothers) from Amhara, 29.0% (2,679 mothers) from SNNPR, 27.1% (2,506 mothers) from Oromia, 7.3% (674 mothers) from Addis Ababa, and 3.0% (276 mothers) from Harar, resulting in a response rate of 98.5%.

#### Factors affecting dietary knowledge, attitude, and practice

Only a few of the included studies reported on the factors influencing knowledge of maternal dietary practice (*n* = 3) and attitude toward it (*n* = 2), while most reported on a variety of variables affecting the dietary practices of mothers during pregnancy.

##### Factors affecting dietary knowledge

These factors were categorized as:

1. Sociodemographic factors such as family size (61), age (61), education (55, 61, 62), income (61, 62), and occupation (55, 61, 62).
2. Gynecological and obstetric variables like parity (55), number of pregnancies (62), gaps or intervals between pregnancy (61), number of antenatal care (ANC) visits (61), chronic diseases or illnesses (61, 62), and body mass index (BMI) (61).
3. Attitude toward maternal nutrition (62).

##### Factors affecting dietary attitude

These were grouped into:

1. Sociodemographic factors, including education (55, 62), income (55, 62), and occupation (62).
2. Gynecological issues such as normality of previous deliveries (62).
3. Knowledge regarding maternal nutrition (62).

##### Factors affecting dietary practice

These factors were thematically classified as:

1. Sociodemographic variables such as family size (51–53, 59, 61, 63, 67), age (56, 57, 61–63), residence (51–53, 57, 64), education (51, 54–56, 58, 60–64, 66, 67), and occupation (52, 56, 57, 60–63, 66).
2. Income and wealth related factors included income (50–56, 58, 60, 62, 63, 67), food security (38, 53, 56, 59), wealth index (38, 57), vegetable production (38), and drinking water (66).
3. Dietary knowledge and information such as ownership of radio (50, 53), dietary knowledge (38, 50–54, 56–58, 60, 62), and nutrition/health information (38, 52, 53, 57, 58, 60, 63, 67).
4. Dietary attitude and intension, such as dietary attitude (52, 53, 55, 57, 58, 60, 62), and intention to take a balanced diet (38).
5. Gynecological and illness experiences included history of illness (50, 61, 65), give birth with a neural tube defect (64), body mass index (61), number of pregnancies (52, 57), stage of pregnancy or gestational age (57, 65), number of live births (60), ANC visits (57, 60, 61, 66), gaps or intervals between pregnancy (54, 56, 57, 60, 61), normality of previous delivery (62), and time to reach health facilities (57).
6. Family support and decision-making, including family or husbands” support (38, 56), and shared household decision making (38).
7. Expectations of nutrition outcomes and habits such as food craving (63), perceived susceptibility to malnutrition (38), perceived severity of malnutrition (38, 65, 66), perceived benefit of good dietary practice (38, 65, 66), perceived self-efficacy or venerability to control malnutrition (65, 66), food restriction or aversion (66), meal frequency (56, 63), and chat chewing (66).

### Quantitative synthesis

#### Levels of dietary knowledge, attitude, and practice

##### Dietary knowledge

**Figure 3:**
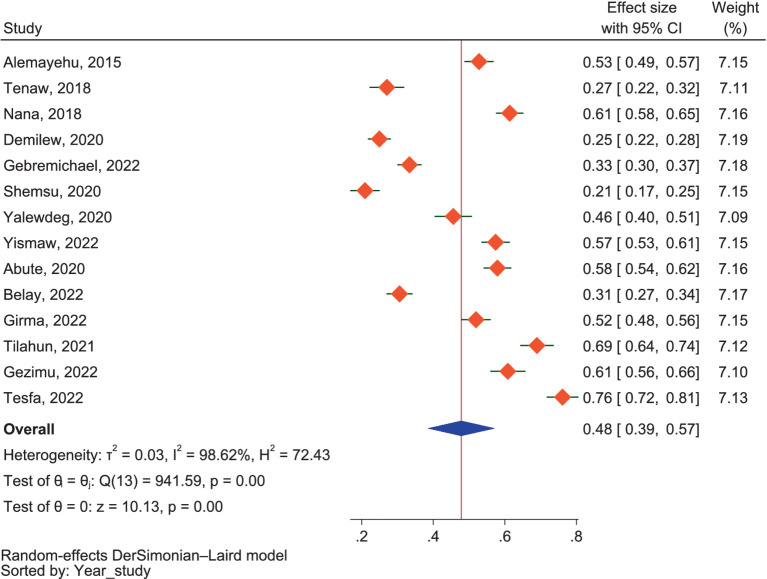
The proportion of good dietary knowledge.

As shown in Figure 3, the pooled results showed that 48.0% of pregnant mothers in Ethiopia had good dietary knowledge regarding dietary practices (95% CI: 39.0–57.0%), with considerable heterogeneity between the included studies (*I*^2^ = 98.62%, *p* < 0.01). The subgroup analysis by region showed that the highest prevalence of good dietary knowledge was reported in SNNPR at 57.0% (95% CI: 50.0–64.0%), followed by Addis Ababa at 52.0% (95% CI: 45.0–59.0%), Amhara at 42.0% (95% CI: 25.0–60.0%), and Oromia at 37.0% (95% CI: 17.0–57.0%) regions. There was no significant subgroup difference between the reports (*p* = 0.17), but there was considerable heterogeneity among the reports of the included studies (*I*^2^ = 98.62%). Despite the lack of significance (*p* = 0.63), the distinction in percentages between community and health facility settings suggests potential variations in dietary knowledge among pregnant mothers based on the study setting. In studies conducted in community settings, the pooled good dietary knowledge report regarding the good dietary practice of pregnant mothers was 45.0% (95% CI: 34.0–57.0%). On the other hand, for studies conducted at health facilities, the pooled good dietary knowledge report was slightly higher at 50.0% (95% CI: 34.0–66.0%).

##### Dietary attitude

**Figure 4:**
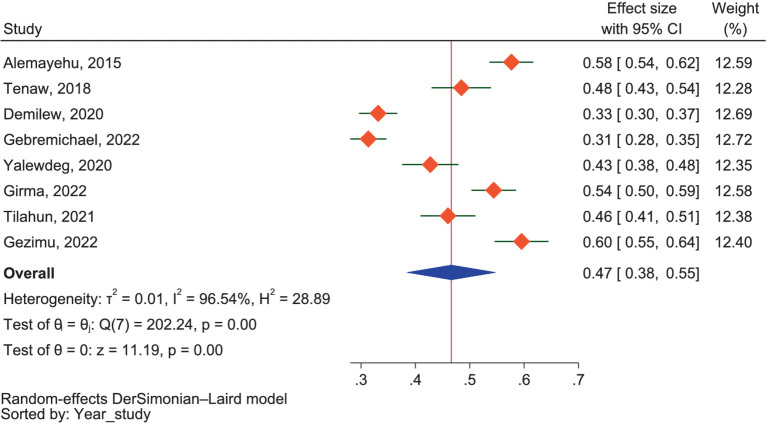
The proportion of favorable attitudes toward good dietary practice.

The combined favorable dietary attitude of pregnant mothers toward good dietary practices was identified as 47.0% (95% CI: 38.0–55.0%), refer to Figure 4. However, it is important to note a considerable level of heterogeneity between studies, as indicated by an *I*^2^ value of 96.54% (*p* < 0.01). The subgroup analysis based on region showed that the highest favorable attitude was 51.0% (95% CI: 43.0–58.0%) in SNNPR, followed by 48.0% (95% CI: 43.0–54.0%) in Addis Ababa, 45.0% (95% CI: 21.0–69.0%) in Amhara, and 31.0% (95% CI: 28.0–35.0%) in Oromia. There were significant subgroup differences between regions regarding the attitude of pregnant mothers (*p* < 0.01). Despite the non-significant result of the difference test (*p* = 0.26), a sub-group analysis based on study setting revealed that the combined result from studies conducted in community settings regarding the favorable attitude of pregnant mothers toward good dietary practices in Ethiopia was 41.0% (95% CI: 25.0–56.0%). In contrast, studies conducted in health facilities reported a slightly higher pooled result of 50.0% (95% CI: 44.0–56.0%).

##### Dietary practice

**Figure 5:**
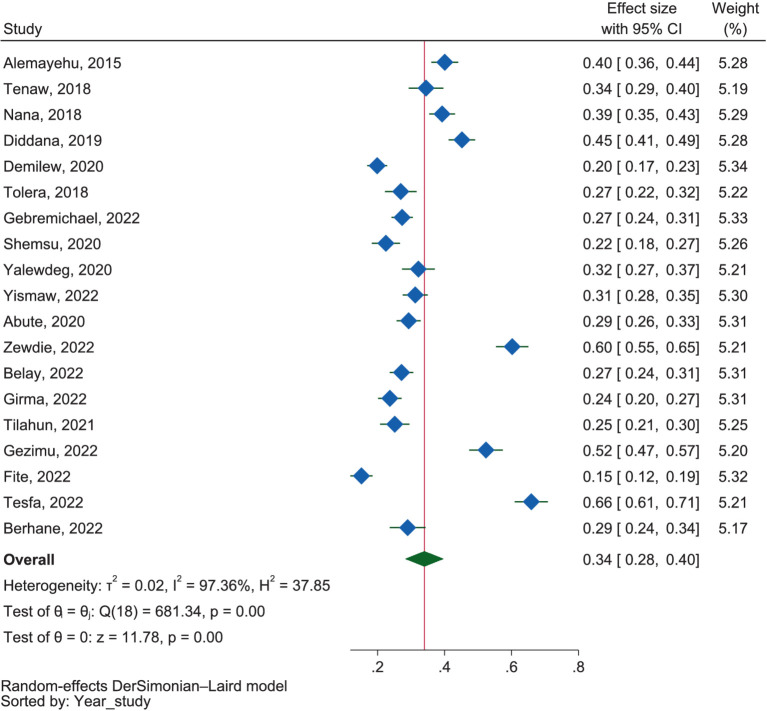
The proportion of good dietary practices.

Figure 5 showed that the combined good dietary practice among pregnant mothers was determined to be 34.0% (95% CI: 28.0–40.0%). However, it is essential to note the presence of considerable heterogeneity between the studies, as indicated by an *I*^2^ value of 97.36% (*p* < 0.01). The subgroup analysis based on region revealed no significant heterogeneity between regions (*p* = 0.13). The highest prevalence of good dietary practice was in Addis Ababa at 50.0% (95% CI: 19.0–81.0%), followed by SNNPR at 37.0% (95% CI: 26.0–49.0%), Amhara at 34.0% (95% CI: 25.0–44.0%), Harar at 29.0% (95% CI: 24.0–34.0%), and Oromia at 25.0% (95% CI: 19.0–30.0%). While the test for the difference did not yield significance (*p* = 0.19), the sub-group analysis based on the study setting revealed notable variations. In the community setting studies, the pooled percentage for good dietary practices was 30.0% (95% CI: 24.0–36.0%), whereas in health facility-based studies, it was comparatively higher at 38.0% (95% CI: 28.0–49.0%).

#### Factors affecting maternal dietary knowledge, attitude, and practice

##### Factors affecting dietary knowledge

Among the sociodemographic factors, education (55, 62), age (61), and income (62) had a positive relationship with good dietary knowledge. However, in the other included studies, income (61) and education (61) had a negative relationship with it, as did family size (61). On the other hand, the influence of occupation (55, 61, 62) on good dietary knowledge was found to be inconsistently reported and was inconclusive. Regarding the gynecological and obstetric variables, parity (55), gravidity (62), number of ANC visits (61), pregnancy interval (61), and chronic diseases or illnesses (61, 62) had a positive relationship with good dietary knowledge, while BMI (61) had the reverse relationship with it. Moreover, good dietary knowledge had a positive relationship with a favorable attitude (62).

##### Factors affecting dietary attitude

The sociodemographic factors such as education (55, 62) and income (55, 62) had a positive relationship with the favorable attitude, while the relationship of occupation (62) with it was found to be inconclusive. Gynecological issues like the normality of previous deliveries (62) and good knowledge (62) were also found to be positively related to it.

##### Factors affecting dietary practice

The analysis of the relationship between dietary practices and associated factors revealed that the findings of the included studies were inconsistent, except for a direct positive relationship between good dietary practices and urban residency, as well as dietary knowledge. However, most studies found an inverse relationship between family size and good dietary practices. Conversely, a majority indicated that good dietary practices improved with higher education levels, higher income, food security, nutrition information, favorable attitudes, and ANC visits.

From the variables listed in Table 3, knowledge, owning a radio, having an illness, residence, attitude, nutrition information, food security, ANC visit, family support, food craving, perceived severity of malnutrition, and perceived benefit of good dietary practice were dichotomized, and the pooled ORs were calculated as shown in the subsequent forest plots.

**Table 3:** The direction of the relationship between the associated factors and the dietary practices of pregnant mothers in Ethiopia.

Themes (variables)	Direction of relationship		
	Positive (+)	Negative (−)	Inconclusive
Sociodemographic variables			
Family size	-	(51–53, 59, 63)	(61, 67)
Mother’s age	(56, 63)	-	(57, 61, 62)
Residence (living in urban area)	(51–53, 57, 64)	-	-
Education level	(51, 55–58, 62, 64, 67)	-	(54, 60, 61, 63, 66)
Occupational status	-	-	(52, 56, 57, 60–63, 66)
Income and wealth related factors			
Income level	(50–54, 60, 62, 63, 67)	(56)	(55, 58)
Food security	(38, 53, 56)	-	(59)
Wealth index	(38)	-	(57)
Vegetable production	(38)	-	-
Clean drinking water	-	(66)	-
Dietary knowledge and information			
Dietary knowledge	(38, 50–54, 56, 57, 60, 62)	-	-
Nutrition information	(38, 52, 53, 57, 58, 67)	(60, 63)	-
Health information	-	(63)	-
Radio ownership	(50, 53)	-	-
Dietary attitude and intension			
Dietary attitude (favorable)	(52, 53, 55, 57, 58, 60, 62)	(55)	-
Intention to take balanced diet	(38)	-	-
Gynecological and illness experiences			
History of illness	(61)	(50, 65)	-
Give birth without neural tube defect	(64)	-	-
Body mass index	-	(61)	-
Number of pregnancies	-	(52)	(57)
Interval or gap between pregnancy	(60)	(54, 56)	(57, 61)
Stage of pregnancy/gestational age	-	-	(57, 65)
Number of live births	-	(60)	-
ANC care visit	(57, 60, 61, 66)	-	-
Time to reach at health facility	-	-	(57)
Normality of previous delivery	-	(62)	-
Family support and decision making			
Family support	(38, 56)	-	-
Household decision making	(38)	-	-
Expectations of nutrition outcomes and habits			
Food craving	(63)	-	-
Meal frequency	(63)	-	-
Susceptibility to malnutrition	(38)	-	-
Severity to malnutrition	(38, 65, 66)	-	-
Benefits of good nutritional practice	(38, 65, 66)	-	-
Food restriction/aversion	-	(66)	-
Venerability to control malnutrition	(65, 66)	-	-
Chat chewing	-	(66)	-

#### Sociodemographic variables

**Figure 6:**
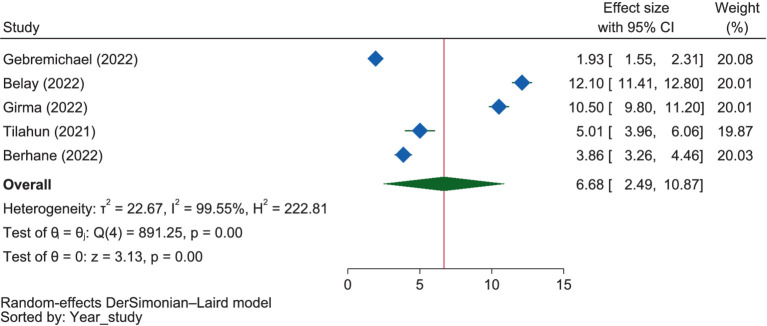
The extent of the influence of residence on good dietary practices.

As shown in Figure 6, pregnant mothers living in urban areas were 6.68 times more likely (OR = 6.68, 95% CI: 2.49–10.87) to have good dietary practices compared to those living in rural milieus. The influence of residence on dietary practice was highest in Amhara, with mothers in urban areas being 12.10 times more likely (OR = 12.10, 95% CI: 11.41–12.80) to have good dietary practices than those in rural areas, followed by SNNPR at 7.77 times (OR = 7.77, 95% CI: 2.39–13.15), Harar at 3.86 times (OR = 3.86, 95% CI: 3.26–4.46), and Oromia at 1.93 times (OR = 1.93, 95% CI: 1.55–2.31).

#### Income and wealth related factors

**Figure 7:**
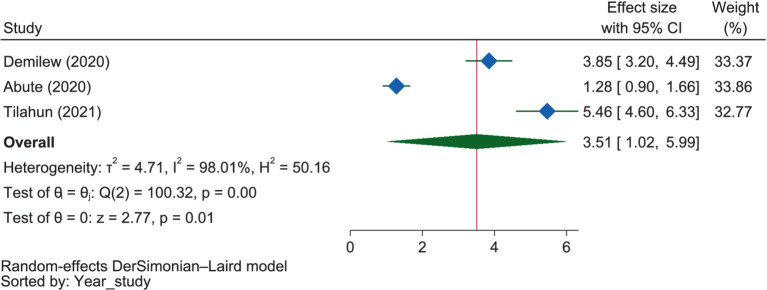
The extent of the influence of food security on good dietary practices.

Figure 7 showed that mothers with sufficient food security were 3.51 times more likely to have good dietary practices than those without (OR = 3.51, 95% CI: 1.02–5.99). The influence of food security on pregnant mothers in SNNPR was not significant (OR = 3.35, 95% CI: −0.75 to 7.45), whereas in Amhara, it significantly influenced mothers’ dietary practices (OR = 3.85, 95% CI: 3.20–4.49). However, no significant heterogeneity between the two regions (*p* = 0.815).

#### Dietary knowledge and information

**Figure 8:**
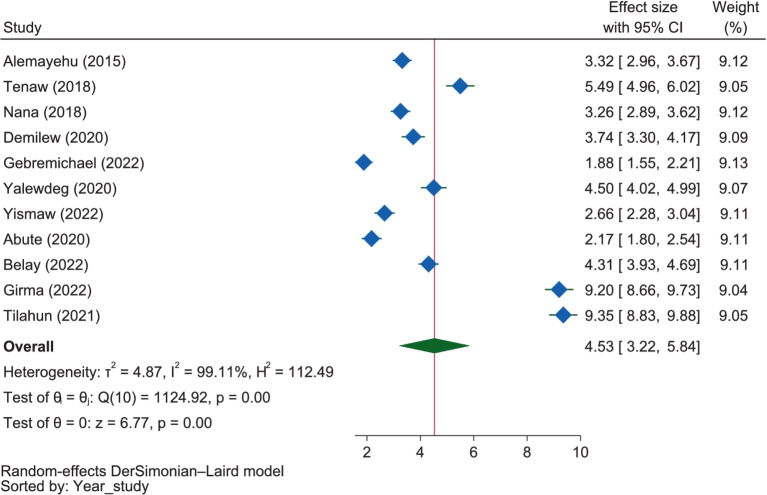
The extent of the influence of knowledge on good dietary practices.

Under this theme, the variables “dietary knowledge,” “ownership of a radio,” and “nutrition information” were reported to be dichotomous. Accordingly, as portrayed in Figure 8, the pooled results showed that pregnant mothers with good dietary knowledge were 4.53 times more likely (OR = 4.53, 95% CI: 3.22–5.74) to have good dietary practices compared to those with poor dietary knowledge. In the SNNPR, mothers with good dietary knowledge were found to be 6.30 times more likely (OR = 6.30, 95% CI: 2.67–9.94) to have good dietary practices than those with poor knowledge, which was the highest likelihood among all subgroups (regions). In Addis Ababa, mothers with good dietary knowledge were 5.49 times more likely (OR = 5.49, 95% CI: 4.96–6.02) to have good dietary practices than those with poor knowledge. In Amhara, mothers with good dietary knowledge were 3.65 times more likely to have good dietary practices than those with poor dietary knowledge (OR = 3.65, 95% CI: 3.16–4.14). Mothers in Oromia with good dietary knowledge were 2.27 times more likely (OR = 2.27, 95% CI: 1.50–3.03) to have good dietary practices than those with poor dietary knowledge, which was the lowest probability among the regions.

**Figure 9:**
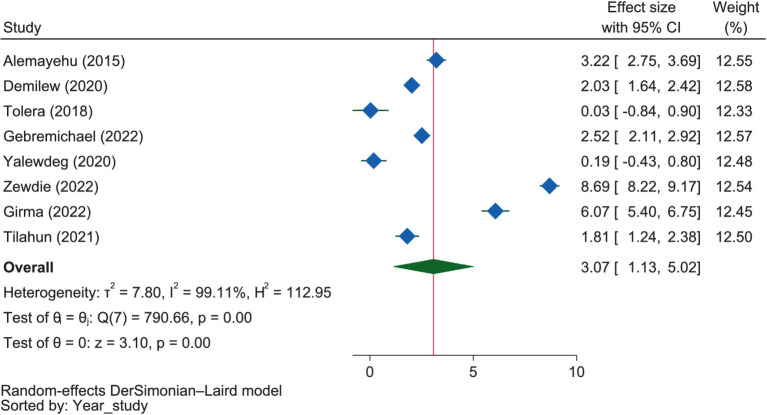
The extent of the influence of nutrition information on good dietary practices.

Pregnant mothers with nutrition information were 3.07 times more likely to have good dietary practices than those without information (OR = OR = 3.07, 95% CI: 1.13–5.02) (Figure 9). Although the influence of nutrition information on good dietary practice was highest in SNNPR at 4.19 times (OR = 4.19, 95% CI: 0.14–8.24), its relationship was not significant, like in Oromia (OR = 1.63, 95% CI: 0.53–2.73), while in Amhara, nutrition information had a significant influence on good dietary practice (OR = 2.61, 95% CI: 1.45–3.78). However, the influence of nutrition information on good maternal dietary practice did not differ significantly among these regions (*p* = 0.294).

**Figure 10:**
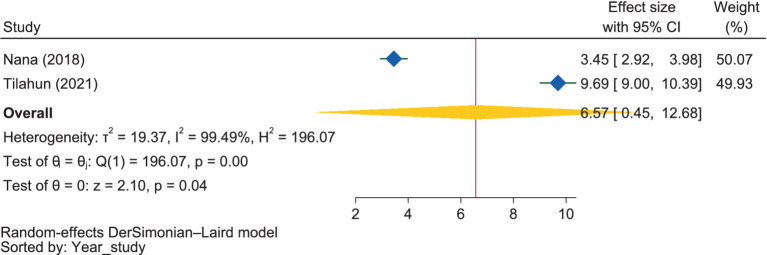
The extent of the influence of radio ownership on good dietary practices.

Regarding radio ownership, although it was not significant, the pooled estimate indicated that pregnant mothers who had a radio in their home were 6.57 times (OR = 6.57, 95% CI: 0.45–12.68) more likely to have good dietary practices than those who did not own a radio (Figure 10). However, the reports from individual studies found that radio ownership significantly influenced dietary practices.

#### Dietary attitude and intension

**Figure 11:**
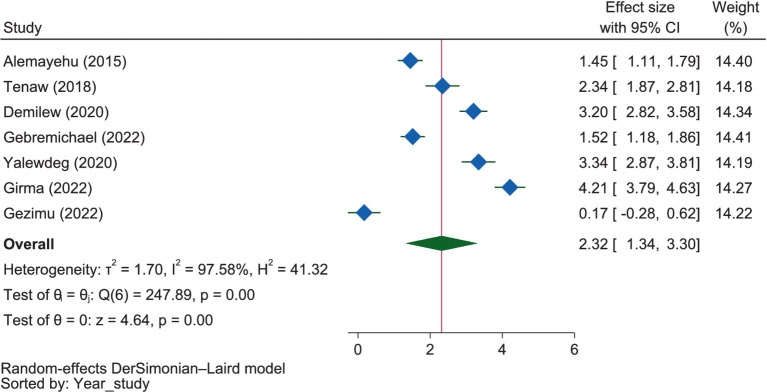
The extent of the influence of attitude on good dietary practices.

The mothers who had a favorable attitude were 2.32 times (OR = 2.32, 95% CI: 1.34–3.30) more likely to have good dietary practices than those who had a poor dietary attitude (Figure 11). Although the relationship was not significant (*p* = 0.037), the subgroup analysis by region showed that mothers with a favorable attitude in Amhara were 2.32 times (OR = 2.32, 95% CI: 0.61–4.04) more likely to have good dietary practices than those with an unfavorable attitude. Similarly, in the SNNPR region, mothers with a favorable attitude were 2.57 times (OR = 2.57, 95% CI: 0.15–5.00) more likely to have good dietary practices compared to those with an unfavorable attitude. A favorable attitude was significantly and positively associated with good dietary practice in Oromia (OR = 1.52, 95% CI: 1.18–1.86) and Addis Ababa (OR = 2.34, 95% CI: 1.87–2.81). The result showed that there was significant heterogeneity between the regions (*p* = 0.037).

#### Gynecological and illness experiences and perceptions

**Figure 12:**
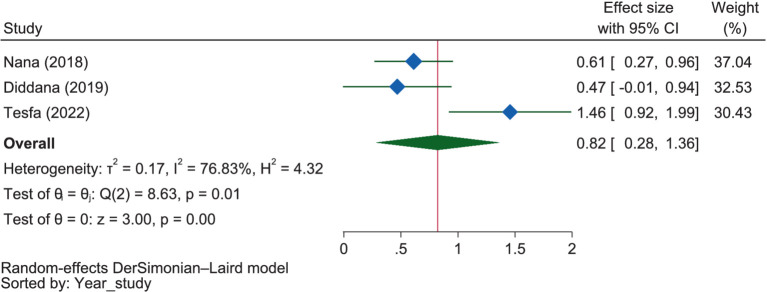
The extent of the influence of illness on good dietary practices.

Illness experience and ANC visits were reported as dichotomous variables within this theme. Though it was not significant, those mothers who experienced illness were 18.0% less likely (OR = 0.82, 95% CI: 0.28–1.36) to have good dietary practices when compared to those who did not experience illness (Figure 12). In considering the subgroup analysis, those who had illness were 1.46 times (OR = 1.46, 95% CI: 0.92–1.99) more likely to have good dietary practice in Amhara, even though it was not significant. This was 2.61 times that of Oromia (OR = 0.56, 95% CI: 0.28–0.84), which was also not significant. There was a significant heterogeneity between these regions regarding the influence of illness on good maternal dietary practice (*p* = 0.004).

**Figure 13:**
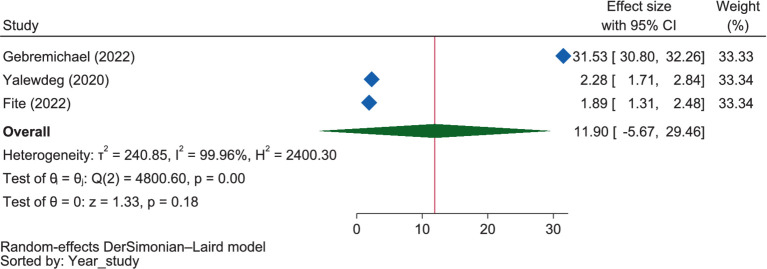
The extent of the influence of the ANC visit on good dietary practices.

Concerning the influence of ANC visits on good dietary practice, the pooled estimate showed that those mothers who had ANC visits were 11.90 times (OR = 11.90, 95% CI: −5.67 to 29.46) more likely to have good dietary practice, though the relationship was not significant (Figure 13). Like the pooled estimate, but unlike in SNNPR (OR = 2.28, 95% CI: 1.71–2.84), the influence of an ANC visit on good dietary practice in Oromia was not significant (OR = 16.71, 95% CI: −12.33 to 45.75).

#### Family support and decision making

**Figure 14:**
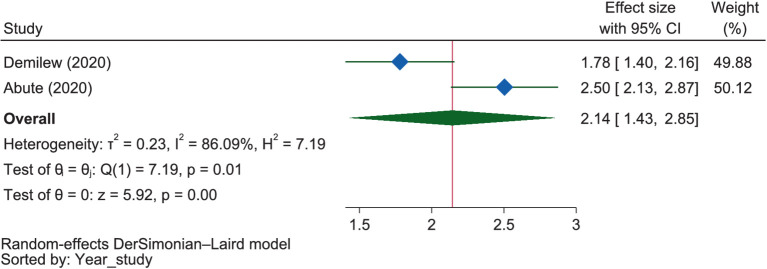
The extent of the influence of family support on good dietary practices.

The pooled estimate from two studies, as presented in Figure 14, indicates that mothers with family support were 2.14 times more likely (OR = 2.14, 95% CI: 1.43–2.85) to engage in good maternal dietary practices than those without support.

#### Psychological and other factors

**Figure 15:**
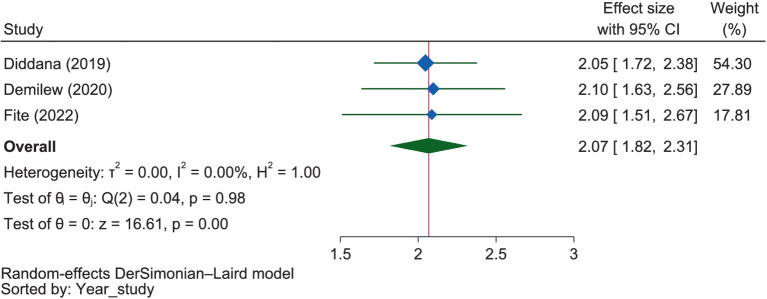
The extent of influence of perceived severity of malnutrition on good dietary practices.

Under this theme, the variables “perceived severity of malnutrition” and “perceived benefit of good dietary practice” were reported by some of the included studies as dichotomous or discrete variables. The pooled estimate regarding the perceived severity of malnutrition showed that pregnant mothers who perceived malnutrition as a severe problem were 2.07 times more likely (OR = 2.07, 95% CI: 1.82–2.31) to have good dietary practices than those who perceived it as less severe (Figure 15). The influence of perceived severity of malnutrition on good maternal dietary practice was comparable in Amhara (OR = 2.06, 95% CI: 1.79–2.33) and Oromia (OR = 2.09, 95% CI: 1.51–2.67), with no significant difference between these regions (*p* = 0.941).

**Figure 16:**
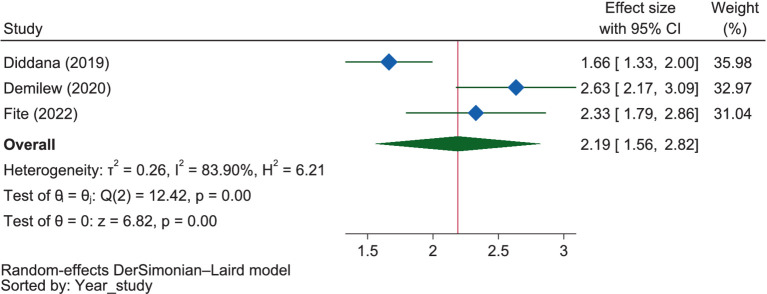
The extent of the influence of the perceived benefits of good nutrition on good dietary practices.

The pooled estimate revealed that mothers with a positive perception of the benefits of good dietary practice during pregnancy were 2.19 times more likely to engage in it (OR = 2.19, 95% CI: 1.56–2.82) compared to those with negative perceptions (Figure 16). This positive perception was associated with a 2.33-fold increase in good dietary practices in Oromia (OR = 2.33, 95% CI: 1.79–2.86) and a 2.14-fold increase in Amhara (OR = 2.14, 95% CI: 1.18–3.09). The report of the studies showed no significant difference between these regions (*p* = 0.730).

### Risk of bias and certainty assessment

#### Knowledge

There was considerable heterogeneity between the included studies regarding dietary knowledge (*I*^2^ = 98.62%). On the other hand, the publication bias among studies was determined using a doi plot, which provided an LFK value of −0.44, indicating the absence of asymmetry. The LFK value of −0.44 suggests that there is no significant asymmetry in the doi plot used to assess publication bias among the studies. The sensitivity analysis conducted on knowledge revealed that seven of the included studies (38, 50, 51, 53, 57, 59, 61) were identified as outliers according to the fixed model effect. However, these studies were not flagged as outliers in the analysis conducted using the random model effect. Consequently, we failed to exclude them from our analysis.

The *I*^2^ statistics indicated considerable heterogeneity between the included studies regarding dietary attitudes (*I*^2^ = 96.54%). However, the analysis conducted to assess publication bias using a doi plot provided an LFK index of 0.64. This index suggests no asymmetry was observed, indicating no publication bias among the included studies. The sensitivity analysis of attitudes revealed that three of the included studies (38, 57, 58) were identified as outliers using the fixed model effect but were not identified as outliers using the random effects model. Consequently, we included them in our analysis to estimate the pooled proportion of favorable dietary attitudes.

**Figure 17:**
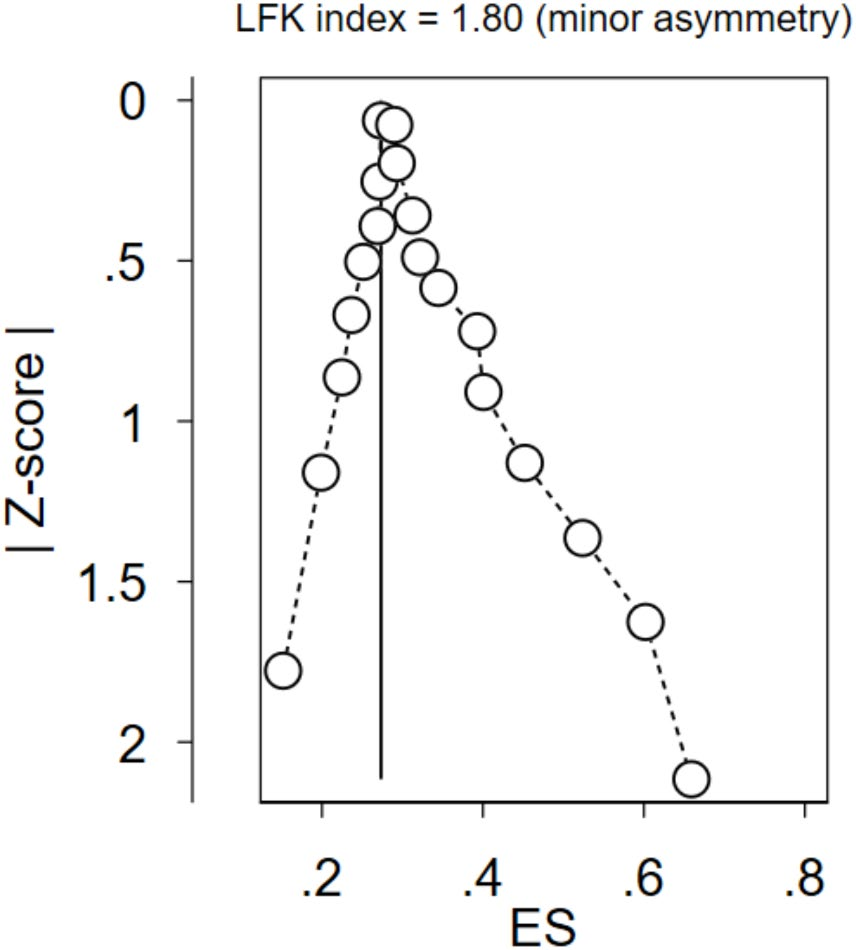
The extent of publication bias among the included studies regarding dietary practice using the LFK value.

Given the considerable heterogeneity observed between studies (*I*^2^ = 97.36%), a random effects model was employed instead of a fixed effects model to account for the influence of potential outlier studies on the overall analysis. In an effort to pinpoint the sources of this heterogeneity, subgroup analyses were conducted based on region and study settings, providing a more nuanced understanding of the variations across different contexts. To assess the potential for publication bias among the studies, the LFK index was calculated (Figure 17), yielding a value of 1.80. This value, indicating minor asymmetry, is considered acceptable within the standards of publication bias assessment.

**Figure 18:**
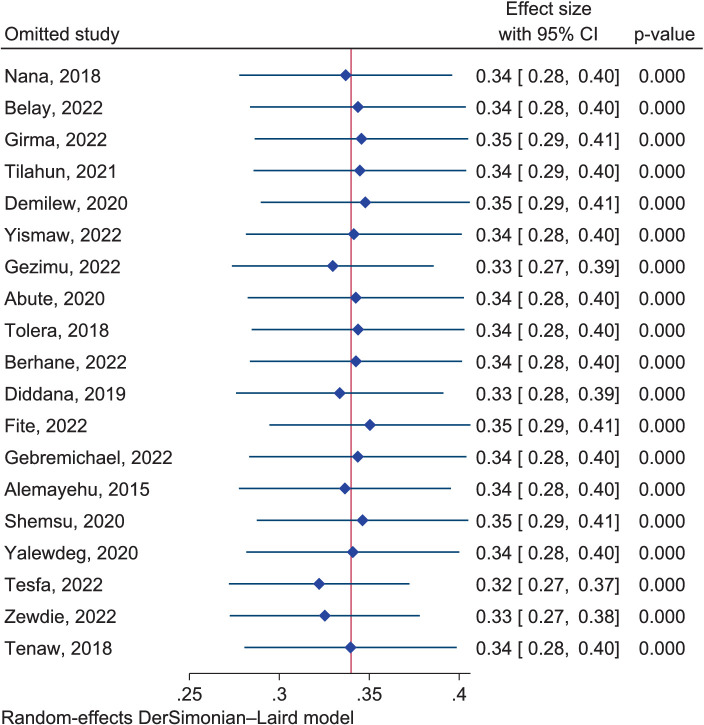
Sensitivity analysis among the included studies regarding dietary practice using a random effects model.

The sensitivity analysis using the fixed effect model showed that four studies were found to be outliers (38, 61, 66, 67). However, after the sensitivity analysis was conducted using the random effect model, no outlier studies were detected (Figure 18). Hence, we failed to exclude those outlier studies.

## Discussion

We comprehensively reviewed the dietary knowledge, attitudes, and practices of pregnant mothers and the associated factors for each. Firstly, we estimated the prevalence of good knowledge, favorable attitudes, and good dietary practices reported by the included studies. Secondly, we qualitatively extracted the variables that affected them. Thirdly, we analyzed the extracted prevalence for each. Finally, we computed the effect sizes from the variables reported as dichotomous data using the reported crosstabs rather than taking the odds ratios directly. As a result, the discussion first addressed the prevalence of good dietary knowledge, favorable attitudes, and good dietary practices, followed by the factors affecting them.

### Prevalence of dietary knowledge, attitude, and practice

Studies conducted in health facility settings reported higher proportions of good dietary knowledge, favorable attitudes, and better dietary practices compared to those conducted in community settings. Whatever it is, the good dietary practice was much lower than both the knowledge of and the attitude toward a good diet, although all were inadequate. This was similar to the findings of a study in Syria, which showed that knowledge, attitude, and practices regarding nutrition and diet during pregnancy were lacking (68). Other studies in Cameroon and India also found that despite satisfactory knowledge and attitudes toward nutrition during pregnancy, significant gaps and difficulties exist in translating them into practice (69, 70), which may be due to confounding factors such as socio-cultural, environmental, economic aspects, and the level of nutrition information, as reported by a study in Mozambique (71).

#### Knowledge

The pooled estimate of our review indicated that the participants’ good dietary knowledge was 48.0% (95% CI: 39.0–57.0%), which was significantly higher than the findings from studies in China at 3.8% (72), Nigeria at 35.0% (73), Benin at 12.0% (74), Iran at 2.5% (75), and Egypt at 4.7% (76). However, it was lower than reports from other studies in Rwanda at 53.6% (77), Iran at 59.1% (78), Nigeria at 86.89% (79), 76.8% (80), and 65.3% (81), Malaysia at 52% (82), and Kenya at 41.8% (83).

#### Attitude

Our review showed that the pooled favorable attitude of pregnant mothers toward good dietary practice in Ethiopia was 47.0% (95% CI: 38.0–55.0%). This result was lower than the reports of studies conducted in China at 91.0% (72), Nigeria at 66.7% (73), 86.89% (79), and 63.27% (81), Iran at 81.10% (78) and 98.2% (75), and Malaysia at 67% (82); however, it was higher than the finding of another study in Rwanda, which showed that the favorable dietary attitude was 32.7% (77).

#### Practice

The pooled result of the dietary practice review among pregnant mothers in Ethiopia showed that 34.0% (95% CI: 28.0–40.0%) had good dietary practices. The dietary practice in our review was lower than the reports of studies in Nigeria at 39.6% (80), Iran at 70.0% (75) and 63.8% (78), and Malaysia at 55.0% (82), but higher than the reports of other studies in Rwanda at 28.2% (77), and China at 16.8% (72).

The differences might be attributed to the sociocultural differences in populations that can lead to differences in food consumption, traditions, and restrictions (84). Cultural beliefs significantly influence food patterns and eating habits (85), particularly in pregnant women (86). In fact, nutrition is one of the 12 domains of culture, with beliefs, values, and food types influencing diet, avoidance, or alteration to align with cultural lifeways (87). This is because individuals’ actions are shaped by socio-cultural environments, policies, and services within families, communities, and countries (88). In contrast, a Nigerian study found that cultural food beliefs do not affect pregnant women’s dietary practices (89), possibly due to some cultural habits that are modifiable (90).

### Factors affecting dietary knowledge, attitude, and practice

The review revealed a complex relationship between knowledge, attitude, and dietary practices. Both knowledge and attitude have a significant impact on dietary practices, with attitudes influencing knowledge and knowledge influencing attitudes. This highlights the importance of considering the mutual influences of knowledge and attitude in promoting positive dietary behaviors in pregnant mothers.

#### Factors affecting dietary knowledge

The factors affecting the dietary knowledge of pregnant mothers include sociodemographic variables such as family size, age, income, education, and occupation; gynecological and obstetric issues like parity, number of pregnancies, intervals between pregnancies, number of ANC visits, pregnancy-related diseases or illnesses, and BMI; and attitudes toward maternal nutrition. Similarly, a study in Australia (91) showed that the dietary knowledge of pregnant mothers was affected by their education level, income, age, stage of pregnancy, language, and possession of a health or nutrition qualification.

Among the sociodemographic factors, two studies (55, 62) have shown a positive relationship between education and good dietary knowledge, i.e., higher levels of education are associated with better dietary knowledge. However, another study (61) reported a negative relationship between education and dietary knowledge, indicating inconsistency in the findings. A single study (61) indicated a positive relationship between age and good dietary knowledge. While a study (62) found a positive association between income and dietary knowledge, similar to the report of a study in Malaysia (92), another study (61) reported a negative relationship. This suggests that the impact of income on dietary knowledge may vary across different populations. One study (61) found that family size had a negative relationship with dietary knowledge. The influence of occupation on dietary knowledge was inconsistently reported and inconclusive across studies (55, 61, 62).

Regarding the gynecological and obstetric variables, two studies (55, 62) showed that parity and gravidity were found to have a positive relationship with good dietary knowledge. A study showed that a higher number of ANC visits and pregnancy interval (61) were associated with better dietary knowledge. The presence of chronic diseases or illnesses during pregnancy was positively associated with good dietary knowledge (61, 62). BMI was found to have an inverse relationship with dietary knowledge in one study (61). Furthermore, good dietary knowledge was positively associated with a favorable attitude toward nutrition (62).

#### Factors affecting dietary attitude

The attitude of pregnant mothers toward good dietary practices is influenced by sociodemographic factors such as education, income, and occupation; gynecological history, including the normality of previous deliveries; and knowledge about maternal nutrition.

Regarding sociodemographic factors, mothers with higher education and income levels tend to have more favorable attitudes toward healthy eating (55, 62). This could be due to several reasons, such as the ability to afford healthier foods and a greater understanding of the link between diet and health. The finding on the relationship between occupation and dietary attitudes was inconclusive (62).

In terms of gynecological factors, women who have had normal deliveries (vaginal deliveries without complications) were more likely to have positive attitudes toward healthy eating than women who have had abnormal deliveries (62). Moreover, women with a good understanding of nutrition were more likely to have positive attitudes toward healthy eating (62). This is likely because knowledge empowers individuals to make informed choices about their diet.

#### Factors affecting dietary practice

The review identified various factors influencing the dietary practices of pregnant mothers, categorized thematically as sociodemographic variables, income and wealth-related factors, dietary knowledge and information, dietary attitude and intention, gynecological and illness experiences and perceptions, family support and decision-making, and psychosocial and other factors. Interestingly, a similar review in Indonesia also highlighted factors affecting the nutritional status of pregnant women, including age, parity, education, knowledge, socio-economic status, infectious diseases, ANC visits, dietary habits, occupation, and origin of residence (93).

### Sociodemographic variables

Under this theme, family size, mother’s age, residence, education, and occupation were found to be affecting the dietary practices of pregnant mothers. Five of the included studies (51–53, 59, 63) found that family size was negatively related to the dietary practices of pregnant mothers. Conversely, a study carried out in Ghana revealed that women with larger households had a high intake of nutrients (94). The age of the mothers was reported by two of the selected studies (56, 63) as a positive predictor of dietary practice. Another systematic review also reported an association between an increase in mothers’ age and healthier dietary changes (95). Eight of the studies (51, 55–58, 62, 64, 67) showed that education level was positively related with the dietary practice. In supporting the result of this review, studies conducted in Kenya noted that as the education attainment of pregnant mothers increased, their good dietary habits were also found to increase (96, 97). Concerning the occupational status, most of the studies’ reports (52, 56, 57, 60–63, 66) were not found to be straightforward enough to confidently determine the relationship between it and the dietary practice. Another review in Indonesia also showed that the dietary practices of pregnant mothers were found to be influenced by their occupational status (93).

Regarding residence, five studies (51–53, 57, 64) consistently reported that living in urban areas was positively related to the good dietary practices of pregnant mothers. The meta-analysis also showed that pregnant mothers living in urban areas were 6.68 times more likely to have good dietary practices compared to those living in rural milieus. A study conducted in China revealed a similar finding, which indicated that women in urban areas had significantly higher reference nutrient intake compared to their counterparts in rural areas (98). In addition, a study conducted in Poland found that the diet of pregnant women from rural areas was characterized by worse quality compared to that of women from urban areas (99). However, a study conducted in South Africa found the opposite result: adults in rural areas have better habits than those in urban areas (100).

### Income and wealth related factors

Income level, food security, wealth index, vegetable production, and availability of clean drinking water were found to be important variables determining dietary practices during pregnancy. The results of this review aligned with findings from studies conducted in Nigeria and Senegal. In Nigeria, a study demonstrated that maintaining adequate nutritional practices during pregnancy was influenced by socio-economic status (101). Similarly, in Senegal, another study revealed a strong relationship between income levels and healthy nutritional practices (102). These congruent findings underscore the significant impact of socio-economic factors, particularly income, on nutritional practices during pregnancy across different geographical regions.

Accordingly, nine of the included studies (50–54, 60, 62, 63, 67) reported that income level had a direct relationship with dietary practices, while a single study (56) reported the opposite. Three of them (38, 53, 56) also found that food security had a positive influence on dietary practices. The meta-analysis also confirmed a positive association between sufficient food security and good dietary practices among mothers. Accordingly, mothers with sufficient food security were 3.51 times more likely to exhibit good dietary practices compared to those without. One study indicated that access to clean drinking water (66) was found to be negatively related to good dietary practices.

### Dietary knowledge and information

Under this category, dietary knowledge, nutrition information, and radio ownership were major factors determining the dietary practices of mothers during gestation. Consequently, most (38, 50–54, 56, 57, 60, 62) of the included studies found that having dietary knowledge was positively associated with dietary practice, which was supported by the result of the meta-analysis in that the mothers with good dietary knowledge were 4.53 times more likely to have good dietary practices compared to those with poor dietary knowledge. On the other hand, a study in Kenya showed that poor knowledge of nutrition could lead to poor dietary practice (103). This means that knowledge about food items was a major factor influencing the maintenance of adequate nutritional practices in pregnancy, as evidenced by a study in Nigeria (101). Indeed, understanding what is eaten allows individuals to make informed decisions about their diets, considering factors such as nutritional value, portion sizes, and overall dietary patterns (104).

Six (38, 52, 53, 57, 58, 67) studies showed a positive association between receiving nutrition information and dietary practice. This evidence was further strengthened by the pooled OR, which showed that pregnant mothers with nutrition information were 3.07 times more likely to have good dietary practices than those without information. Similarly, a study in Indonesia found that inadequate nutritional information was a major detrimental factor for good dietary practice during pregnancy (105). However, two of the included studies (60, 63) reported that nutrition information was inversely related to dietary practice.

Two (50, 53) studies revealed that owning a radio was positively associated with dietary practice, i.e., though it was not significant, pregnant mothers who had a radio in their home were 6.57 times more likely to have good dietary practices than those who did not own a radio. Another study showed that a common source of information on nutrition during pregnancy were health professionals (106).

### Dietary attitude and intension

The review revealed that, as shown by seven of the included studies, having a positive attitude (52, 53, 55, 57, 58, 60, 62) and dietary intention (38) were found to be positively related to the dietary practices of pregnant mothers. This was further supported by the meta-analysis, which found that mothers who had a favorable attitude were 2.32 times more likely to have good dietary practices than those with an unfavorable attitude. In contrast, one (55) of the included studies reported that a positive attitude was negatively related to the dietary practice, which might be due to ignorance, as evidenced by a study in Nigeria (101).

### Gynecological and illness experiences

Under this theme, ANC visits (57, 60, 61, 66) were found to be positively related to dietary practices, which was supported by the result of the pooled OR, which showed those mothers who had ANC visits were 11.90 times more likely to have good dietary practices, though the relationship was not significant. This might be due to the fact that ANC coverage is crucial for access to healthcare during pregnancy, including dietary education, which can benefit pregnant women through interventions vital to their health and that of their infants. Receiving antenatal care at least four times can thus increase the likelihood of effective maternal health interventions, which is part of the Sustainable Development Strategy (2016–2030) for Women’s, Children’s, and Adolescents’ Health (107).

Two of the included studies showed that a history of illness (50, 65) was negatively related to the dietary practice, while one study (61) found the opposite. Similarly, though it was not significant, the pooled OR implied that those mothers who experienced illness were 18.0% less likely to have good dietary practices when compared to those who did not experience illness. This might be because illness can significantly impact individuals and communities, affecting household food availability and dietary practices. During illness, economic productivity may decrease, leading to limited food availability (108). This can result in poor dietary practices, particularly among pregnant women, who may reduce meal frequency as a coping mechanism for food insecurity (109), which can negatively impact the health and well-being of both the mother and her unborn child (110).

In the same manner, two of the included studies (54, 56) found that the interval or gap between pregnancy was found to be negatively related to dietary practice, but one (60) found the reverse. Another systematic review also revealed inconsistent findings regarding the effect of birth spacing on the dietary practices of pregnant mothers (111). However, it is generally agreeable that adequate birth spacing during pregnancy is beneficial for maternal nutrition, as it allows mothers time to recover and understand proper nutrition, while shorter intervals may hinder optimal dietary practices due to inadequate recovery time and competing demands. Hence, proper timing and spacing can reduce mortality risk in children under five by 25% (112).

The number of live births (60) and previous normal deliveries (62) were also found to be negatively related to the dietary practice. This might be due to the fact that mothers who previously experienced normal live births through normal deliveries might not be worried about their maternal nutrition, maintain the status quo, and expect the same positive outcome in their future pregnancy.

### Family support and decision making

Family support (38, 56) and mothers’ participation (38) in household decision-making were found to be positively related to pregnant mothers’ dietary practices. The pooled quantitative estimate from two studies similarly indicated that mothers with family support were 2.14 times more likely to engage in good maternal dietary practices than those without support. This evidence was supported by a study in Nigeria, which found that good dietary practice in pregnancy was hindered by a lack of husband support (101). This might be because individuals’ decisions are influenced by their social and economic groups, physical and market environments, and public and private services and policies, which are shaped by their families, communities, and countries (88).

### Expectations of nutrition outcomes and habits

Under this theme, food craving and meal frequency (63), perceived susceptibility to malnutrition (38), perceived severity to malnutrition (38, 65, 66), perceived benefits of good nutritional practice (38, 65, 66), and venerability to control malnutrition (65, 66) were positively related to the good dietary practice, while food restriction/aversion and chat chewing (66) were negatively related to it. Accordingly, pregnant mothers who perceived malnutrition as a severe problem were 2.07 times more likely to have good dietary practices than those who perceived it as less severe. Similarly, mothers with a positive perception of the benefits of good dietary practice during pregnancy were 2.19 times more likely to engage in it compared to those with negative perceptions.

According to a study in Pakistan, food aversions and cravings were reported to hinder healthy dietary intake during pregnancy (113). Another study in Ghana also showed that pregnant women are forbidden from eating nutritious foods due to these issues and traditional beliefs (114). For instance, a study in Brazil confirmed that the majority of pregnant mothers perceive their diets as unhealthy (115). On the other hand, similar to the findings of the current review, a study in Indonesia revealed that there are factors beyond those widely studied, such as self-efficacy (93).

## Limitations

Due to the lack of comparator reviews, we discussed the results of our systematic and meta-analyses against original studies conducted in various other countries. Moreover, we calculated the crude ORs using cross-tabulations instead of using the adjusted ORs reported by the included studies, due to errors found in some authors’ calculations and interpretations of the crude ORs that could render the adjusted ORs incorrect.

## Policy and practical implications

Ensuring the availability, accessibility, safety, and quality of nutritious foods is essential for fostering a productive workforce, enhancing longevity, improving livelihoods, and boosting innovative capacity. This, in turn, contributes to rapid economic, social, and sustainable development. Achieving these goals involves promoting healthy living, enhancing knowledge of nutrient-rich foods, improving food utilization, ensuring food safety and quality, minimizing food and nutrient losses, developing food and nutrition emergency preparedness, and building resilience capacity (116). To do so, our review underscores the urgency of nutrition education interventions (117).

## Direction to future research

Future research in the realm of maternal nutrition during pregnancy could explore several critical thematic areas to enhance our understanding and guide interventions. It is crucial to investigate the impact of intrapersonal physiological factors like nausea and vomiting, as well as interpersonal factors, on healthy eating during pregnancy (118). Further exploration of biological determinants such as hunger, appetite, and taste (90, 119), along with physical determinants like access, skills, and time, is warranted. Examining social determinants, including social class, culture, and social context, is essential for a comprehensive understanding of maternal nutrition. Additionally, delving into psychological determinants such as mood, stress, and guilt (90) can provide valuable insights.

## Conclusion

The prevalence of the pooled good dietary knowledge, the favorable dietary attitude toward it, and the good dietary practice were found to be suboptimal. To the worst, the good dietary practice was lower than both the good knowledge of nutrition and the favorable attitude toward it, though all were found to be below 50.0%. Mothers who had good knowledge and favorable attitude were found to have better dietary practice, which was a paradox when considering the lower good dietary practice compared to a relatively higher good dietary knowledge, and favorable attitude, which might be associated with other confounding factors such as sociodemographic variables, income and wealth related factors, nutrition information, dietary intension, gynecological and illness experiences, family support and decision-making, and expectations of nutrition outcomes and habits; of which sociodemographic and gynecological issues also found to affect those dietary knowledge and attitude, which by themselves were found to have relationships.

## Data availability statement

The original contributions presented in the study are included in the article/Supplementary material; further inquiries can be directed to the corresponding author.

## Author contributions

EB: Conceptualization, Data curation, Formal analysis, Investigation, Methodology, Project administration, Resources, Software, Supervision, Validation, Visualization, Writing – original draft, Writing – review & editing. EY: Conceptualization, Formal analysis, Methodology, Project administration, Supervision, Validation, Visualization, Writing – review & editing. TG: Conceptualization, Formal analysis, Methodology, Resources, Supervision, Validation, Visualization, Writing – review & editing. AM: Conceptualization, Project administration, Resources, Supervision, Validation, Visualization, Writing – review & editing. NM: Conceptualization, Project administration, Resources, Supervision, Validation, Visualization, Writing – review & editing.
