# Supplementary material for: Dietary knowledge, attitude, practice, and associated factors among pregnant mothers in Ethiopia: a systematic review and meta-analysis
Source: Front Public Health. 2024 Sep 11;12:1393764. doi: 10.3389/fpubh.2024.1393764 (PMC11425043; doi:10.3389/fpubh.2024.1393764)
Supplement: Supplementary file 2 [file Table_2.DOCX]

**Supplementary file 2. Database search strategy**

1. **PubMed (n = 231):**

Search: **((((((Dietary practice) OR (Nutrition)) OR (Knowledge)) OR (Attitude)) AND (Associated factor)) AND (Pregnant mother)) AND (Ethiopia)**

((("diet"[MeSH Terms] OR "diet"[All Fields] OR "dietary"[All Fields] OR "dietaries"[All Fields]) AND ("practicability"[All Fields] OR "practicable"[All Fields] OR "practical"[All Fields] OR "practicalities"[All Fields] OR "practicality"[All Fields] OR "practically"[All Fields] OR "practicals"[All Fields] OR "practice"[All Fields] OR "practice s"[All Fields] OR "practiced"[All Fields] OR "practices"[All Fields] OR "practicing"[All Fields])) OR ("nutrition s"[All Fields] OR "nutritional status"[MeSH Terms] OR ("nutritional"[All Fields] AND "status"[All Fields]) OR "nutritional status"[All Fields] OR "nutrition"[All Fields] OR "nutritional sciences"[MeSH Terms] OR ("nutritional"[All Fields] AND "sciences"[All Fields]) OR "nutritional sciences"[All Fields] OR "nutritional"[All Fields] OR "nutritionals"[All Fields] OR "nutritions"[All Fields] OR "nutritive"[All Fields]) OR ("knowledge"[MeSH Terms] OR "knowledge"[All Fields] OR "knowledge s"[All Fields] OR "knowledgeability"[All Fields] OR "knowledgeable"[All Fields] OR "knowledgeably"[All Fields] OR "knowledges"[All Fields]) OR ("attitude"[MeSH Terms] OR "attitude"[All Fields] OR "attitudes"[All Fields] OR "attitude s"[All Fields])) AND (("associate"[All Fields] OR "associated"[All Fields] OR "associates"[All Fields] OR "associating"[All Fields] OR "association"[MeSH Terms] OR "association"[All Fields] OR "associations"[All Fields]) AND ("factor"[All Fields] OR "factor s"[All Fields] OR "factors"[All Fields])) AND (("pregnant"[All Fields] OR "pregnants"[All Fields]) AND ("mother s"[All Fields] OR "mothered"[All Fields] OR "mothers"[MeSH Terms] OR "mothers"[All Fields] OR "mother"[All Fields] OR "mothering"[All Fields])) AND ("ethiopia"[MeSH Terms] OR "ethiopia"[All Fields] OR "ethiopia s"[All Fields])

**Translations**

**Dietary:** "diet"[MeSH Terms] OR "diet"[All Fields] OR "dietary"[All Fields] OR "dietaries"[All Fields]

**Practice:** "practicability"[All Fields] OR "practicable"[All Fields] OR "practical"[All Fields] OR "practicalities"[All Fields] OR "practicality"[All Fields] OR "practically"[All Fields] OR "practicals"[All Fields] OR "practice"[All Fields] OR "practice's"[All Fields] OR "practiced"[All Fields] OR "practices"[All Fields] OR "practicing"[All Fields]

**Nutrition:** "nutrition's"[All Fields] OR "nutritional status"[MeSH Terms] OR ("nutritional"[All Fields] AND "status"[All Fields]) OR "nutritional status"[All Fields] OR "nutrition"[All Fields] OR "nutritional sciences"[MeSH Terms] OR ("nutritional"[All Fields] AND "sciences"[All Fields]) OR "nutritional sciences"[All Fields] OR "nutritional"[All Fields] OR "nutritionals"[All Fields] OR "nutritions"[All Fields] OR "nutritive"[All Fields]

**Knowledge:** "knowledge"[MeSH Terms] OR "knowledge"[All Fields] OR "knowledge's"[All Fields] OR "knowledgeability"[All Fields] OR "knowledgeable"[All Fields] OR "knowledgeably"[All Fields] OR "knowledges"[All Fields]

**Attitude:** "attitude"[MeSH Terms] OR "attitude"[All Fields] OR "attitudes"[All Fields] OR "attitude's"[All Fields]

**Associated:** "associate"[All Fields] OR "associated"[All Fields] OR "associates"[All Fields] OR "associating"[All Fields] OR "association"[MeSH Terms] OR "association"[All Fields] OR "associations"[All Fields]

**Factor:** "factor"[All Fields] OR "factor's"[All Fields] OR "factors"[All Fields]

**Pregnant:** "pregnant"[All Fields] OR "pregnants"[All Fields]

**Mother:** "mother's"[All Fields] OR "mothered"[All Fields] OR "mothers"[MeSH Terms] OR "mothers"[All Fields] OR "mother"[All Fields] OR "mothering"[All Fields]

**Ethiopia:** "ethiopia"[MeSH Terms] OR "ethiopia"[All Fields] OR "ethiopia's"[All Fields]

1. **Google scholar (n = 179)** **– using “Perish or Publish” software:**

**Dietary practice associated factors pregnant mothers Ethiopia**

*Publish or Perish 8.9.4554.8721 (basic report)
WinPosix (x64) edition, running on WinPosix 10.0.19045 (x64)*

**Search terms**

**Keywords:** Dietary practice associated factors pregnant mothers Ethiopia
**Years:** all
**Other options:** include citations; include patents

**Data retrieval**

**Data source:** Google Scholar
**Search date:** 2024-01-03 11:04:54 +00300
**Cache date:** 2024-01-03 08:10:59 +00300
**Search result:** [0] No error

***Important:*** *This data source provides only abbreviated data. Any ellipses (... marks) shown in this report originate with the data source; they are NOT caused by subsequent processing in Publish or Perish.*

**Metrics**

**Reference date:** 2024-01-03 08:10:59 +00300
**Publication years:** 1998-2023
**Citation years:** 26 (1998-2024)
**Papers:** 179
**Citations:** 7727
**Citations/year:** 297.19 (acc1=159, acc2=129, acc5=84, acc10=45, acc20=8)
**Citations/paper:** 38.64
**Citations/author:** 2815.72
**Papers/author:** 73.72
**Authors/paper:** 3.27/3.0/3 (mean/median/mode)
**Age-weighted citation rate:** 1161.91 (sqrt=34.09), 414.73/author
**Hirsch h-index:** 53 (a=2.75, m=2.04, 6003 cites=77.7% coverage)
**Egghe g-index:** 84 (g/h=1.58, 7080 cites=91.6% coverage)
**PoP hI,norm:** 31
**PoP hI,annual:** 1.19
**Fassin hA-index:** 16

1. **Scopus (n =** **24) – using “perish or publish” software:**

**Dietary practice associated factors pregnant mothers Ethiopia**

*Publish or Perish 8.9.4554.8721 (basic report)
WinPosix (x64) edition, running on WinPosix 10.0.19045 (x64)*

**Search terms**

**Keywords:** Dietary practice associated factors pregnant mothers Ethiopia
**Years:** all

**Data retrieval**

**Data source:** Scopus
**Search date:** 2024-01-03 10:48:43 +00300
**Cache date:** 2024-01-03 07:48:46 +00300
**Search result:** [0] No error

***Important:*** *This data source returns only one author per article; this affects the calculation of per-author metrics.*

**Metrics**

**Reference date:** 2024-01-03 07:48:46 +00300
**Publication years:** 2015-2023
**Citation years:** 9 (2015-2024)
**Papers:** 24
**Citations:** 436
**Citations/year:** 48.44 (acc1=14, acc2=12, acc5=6, acc10=0, acc20=0)
**Citations/paper:** 18.17
**Citations/author:** 436.00
**Papers/author:** 24.00
**Authors/paper:** 1.00/1.0/1 (mean/median/mode)
**Age-weighted citation rate:** 71.47 (sqrt=8.45), 71.47/author
**Hirsch h-index:** 9 (a=5.38, m=1.00, 402 cites=92.2% coverage)
**Egghe g-index:** 20 (g/h=2.22, 436 cites=100.0% coverage)
**PoP hI,norm:** 9
**PoP hI,annual:** 1.00
**Fassin hA-index:** 6

1. **Research4Life (R4L) - Hinari (N= 192):**

**Search:** ((Dietary practice) OR (Knowledge) OR (Attitude)) AND (Pregnant mother) AND (Associated factor) AND (Ethiopia)

**Date:** 2024-01-03 (03 January 2024)

**Selected by:**

- **Accessibility:** Full text online, open access, scholarly and per-reviewed
- **Content Type:** Journal article
- **Publication date:** 15 years
- **Discipline:** Public health and nursing
- **Subject terms:** Dietary practice, pregnant women
- **Language:** English
